# Comparison of colistin-induced nephrotoxicity between two different formulations of colistin in critically ill patients: a retrospective cohort study

**DOI:** 10.1186/s13756-021-00977-w

**Published:** 2021-07-30

**Authors:** Jia-Yih Feng, Yi-Tzu Lee, Sheng-Wei Pan, Kuang-Yao Yang, Yuh-Min Chen, David Hung-Tsang Yen, Szu-Yuan Li, Fu-Der Wang

**Affiliations:** 1 Department of Chest Medicine, Taipei Veterans General Hospital, No. 201, Sec. 2, Shipai Road, Taipei, Taiwan; 2 School of Medicine, National Yang Ming Chiao Tung University, No. 155, Sec. 2, Linong Street, Taipei, Taiwan; 3 Institute of Emergency and Critical Care Medicine, National Yang Ming Chiao Tung University, No. 155, Sec. 2, Linong Street, Taipei, Taiwan; 4 Department of Emergency Medicine, Taipei Veterans General Hospital, No. 201, Sec. 2, Shipai Road, Taipei, Taiwan; 5 Institute of Public Health, National Yang Ming Chiao Tung University, No. 155, Sec. 2, Linong Street, Taipei, Taiwan; 6 Division of Nephrology, Department of Medicine, Taipei Veterans General Hospital, No. 201, Sec. 2, Shipai Road, Taipei, Taiwan; 7 Division of Infectious Diseases, Department of Medicine, Taipei Veterans General Hospital, No. 201, Sec. 2, Shipai Road, Taipei, 11217 Taiwan

**Keywords:** Colistin, Nephrotoxicity, Acute kidney injury, Formulation, Mortality

## Abstract

**Background:**

Colistin is widely used in the treatment of nosocomial infections caused by carbapenem-resistant gram-negative bacilli (CR-GNB). Colistin-induced nephrotoxicity is one of the major adverse reactions during colistin treatment. Comparisons of colistin-induced nephrotoxicity between different formulations of colistin are rarely reported.

**Methods:**

In this retrospective cohort study, we enrolled intensive care unit–admitted patients if they had culture isolates of CR-GNB and underwent intravenous treatment with colistin. The occurrence of acute kidney injury (AKI) during intravenous treatment with colistin was recorded. The occurrence of colistin-induced nephrotoxicity was compared between two formulations of colistin, Locolin®, and Colimycin®. Treatment outcomes associated with the occurrence of colistin-induced nephrotoxicity were also investigated.

**Results:**

Among 195 patients, 95 who were treated with Locolin® and 100 who were treated with Colimycin® were included for analysis. Patients treated with Locolin® had a higher rate of occurrence of stage 2 (46.3% vs. 32%, p = 0.040) and stage 3 (29.5% vs. 13%, p = 0.005) AKI than did those treated with Colimycin®. In multivariate analysis, the presence of septic shock (adjusted odds ratio [aOR] 2.17, 95% confidence interval [CI] 1.10–4.26) and inappropriate colistin dosage (aOR 2.52, 95% CI 1.00–6.33) were clinical factors associated with colistin-induced nephrotoxicity. Treatment with Colimycin® was an independent factor associated with a lower risk of colistin-induced nephrotoxicity (aOR 0.37, 95% CI 0.18–0.77). The mortality rate was comparable between patients with and without colistin-induced nephrotoxicity.

**Conclusions:**

The risk of colistin-induced nephrotoxicity significantly varied in different formulations of colistin in critically ill patients. Colistin-induced nephrotoxicity was not associated with increased mortality rate.

**Supplementary Information:**

The online version contains supplementary material available at 10.1186/s13756-021-00977-w.

## Background

The emergence of multidrug-resistant organisms (MDRO) in nosocomial infections is a growing threat to the global health care system. Carbapenem-resistant gram-negative bacilli (CR-GNB), such as CR-*Acinetobacter baumannii* complex (CRAB), CR-Enterobacterales (CRE), and CR-*Pseudomonas aeruginosa* (CRPA), are common MDRO in nosocomial infections and are associated with high morbidity and mortality rates [1–3]. Colistin is one of the key agents used in the treatment of CR-GNB-induced nosocomial infections, especially hospital-acquired pneumonia [4].

Colistin is a polypeptide antibiotic and acts specifically against gram-negative bacteria [5]. Despite the increasing use of colistin in the treatment of nosocomial infection, the use of intravenous colistin is frequently limited by its adverse reactions, including nephrotoxicity and neurotoxicity [6–9]. It is proposed that the interaction between colistin and phospholipids in the cell membrane can lead to high membrane permeability of tubular epithelial cells and acute tubular necrosis [10]. Colistin-induced nephrotoxicity is characterized by decreased creatinine clearance, proteinuria, cylindruria, or oliguria, and it usually occurs in the first 5–7 days of treatment [11–14]. If discontinued early, then acute kidney injury (AKI) is mostly alleviated within 10 days from discontinuation [13].

Several clinical factors such as old age, underlying comorbidities, concomitant nephrotoxins, higher colistin dose, and presence of septic shock have been proposed to be related to colistin-induced nephrotoxicity [15–18]. However, the risk of nephrotoxicity based on different brands of colistin has rarely been compared thus far. Previous in vitro studies have demonstrated that colistimethate from different brands of colistin may have different structural profiles and generates different range of exposure to colistin [19]. These differences may lead to variations in toxicodynamic effects in humans, which is an important issue in critically ill patients. In the present study, we hypothesized that the risk of nephrotoxicity could be different based on different formulations of colistin. To test our hypothesis, we conducted a retrospective study wherein we enrolled critically ill patients who were treated with two different formulations of intravenous colistin. We aimed to compare differences in the occurrence of AKI between patients who received two different formulations of intravenous colistin. The clinical factors associated with AKI and the effect of AKI on treatment outcomes of critically ill patients were investigated. We also performed high-performance liquid chromatography (HPLC) to determine the difference in the composition of the two colistin formulations.

## Methods

### Patients and settings

This was a retrospective study conducted in a referral medical center in Taiwan. Between January 2016 and October 2018, patients admitted in the intensive care unit (ICU) were enrolled if CR-GNB, including CRAB, CRE, and CRPA, isolates was cultured from their clinical specimens and if they received intravenous colistin for ≥ 48 h. In patients receiving more than one treatment course, only the first treatment course was included for analysis. Patients were excluded if they were aged < 20 years old, had a history of end-stage renal disease (ESRD), were under regular dialysis during initiation of intravenous colistin, missing data of baseline creatinine and at least three follow-up creatinine, and was treated with different brands of colistin in one treatment course. The study was approved by the Institutional Review Board of Taipei Veterans General Hospital, and the need for informed consent was waived (IRB No.: 2019-11-009AC).

### Data collection

Data of demographic characteristics (age, sex, body mass index, and smoking status) and underlying comorbidities (diabetes, malignancies, renal insufficiency, chronic liver disease, and heart failure) were obtained from hospital chart review. Infection sources were determined according to the site of specimen collection. In patients with multiple samples containing CR-GNB, the sample that was obtained closest to the date of prescription of colistin was used to define infection source. Disease severity was evaluated by the Acute Physiology and Chronic Health Evaluation (APACHE) II score on the day of ICU admission. The presence of respiratory failure and septic shock (defined per SEPSIS-2 criteria) on the day of specimen collection was also recorded.

### Carbapenem and colistin resistance determination

Bacteria were phenotypically identified using the Vitek 2 system (bioMérieux, Marcy l’Etoile, France). The minimum inhibitory concentration (MIC) of carbapenems was determined using the broth microdilution method, according to the CLSI guidelines [20]. Carbapenem resistance was defined as resistance to imipenem or meropenem (imipenem or meropenem MIC ≥ 4 mg/L for Enterobacterales and MIC ≥ 8 mg/L for *Pseudomonas aeruginosa* and *Acinetobacter* spp.) [20]. Colistin MICs were determined by broth microdilution as recommended by the joint CLSI-EUCAST Polymyxin Breakpoints Working Group [21].

### Intravenous colistin administration and concomitant nephrotoxins

All the enrolled patients received intravenous colistin for ≥ 48 h. Intravenous colistin was initiated within seven days of CR-GNB cultured from clinical specimens. For patients with multiple treatment courses of intravenous colistin, only the first treatment course was recorded. Two formulations of colistin, namely, Locolin® (Gentle, Taiwan) and Colimycin® (T.T.Y., Taiwan), were available during the study period. Both the formulations were supplied as 66.8 mg of colistin base activity per vial, which was considered equivalent to 2 million IU of sodium colistin methane sulfonate. The recommended loading and maintenance dosing of intravenous colistin was based on previous suggestion [22], and adjusted based on body weight and renal function (Additional file 1: Table 1). Estimated glomerular filtration rate (eGFR) was estimated using the Chronic Kidney Disease Epidemiology Collaboration equation [23]. Maintenance dose of intravenous colistin above the recommended dose was determined as inappropriate colistin dosage.

Concomitant nephrotoxins, including aminoglycoside, vancomycin, and intravenous contrast agent, that were administered within 28 days of colistin treatment were also recorded.

### Analysis of colistin methane sulfonate (CMS) by HPLC

The composition of Locolin® and Colimycin® was analyzed by HPLC. The CMS reference standard was obtained from U.S. Pharmacopeia (Rockville, MD, USA). The 600/717 HPLC system (Waters Corporation, Milford, Massachusetts, USA) consisted of a quaternary pump with an offline vacuum degasser and an autosampler with an injection capacity of 100 μL. Chromatographic separation of Colistin A and Colistin B was achieved on the C18 column (LiChrospher® 100, LiChroCART® 250–4, 150 mm × 4.6 mm i.d.).

### Outcome definition

The primary outcome evaluated in the present study was a comparison of the difference in the risk of newly developed AKI between the two formulations of colistin during intravenous colistin treatment. Serum creatinine levels were recorded at baseline (day 1 of intravenous colistin administration) and thereafter until the end of colistin treatment or death. AKI was determined based on the definition of KDIGO recommendation by creatinine criteria [24]. The occurrence of stage 1 (with 1.5- to 1.9-fold increase or ≥ 0.3 mg/dL increase in the serum creatinine level), stage 2 (with 2- to 2.9-fold increase in the serum creatinine level than that at baseline), and stage 3 (≥ threefold increase in the creatinine level than that at baseline, or ≥ 4.0 mg/dL) AKI, and newly onset dialysis was recorded. Other clinical factors associated with the development of AKI were also investigated.

We also compared clinical outcomes, including mechanical ventilator days, ICU stays, hospital stays, and all-cause mortality at day 28 and on discharge, between patients with and without AKI development.

### Statistical analysis

Statistical analyses were performed using SPSS, version 20.0, software (SPSS, Inc., Chicago, IL, USA). Participants were categorized into the Locolin® and Colimycin® groups and analyzed accordingly. Continuous variables such as APACHE II and hospital stays between subgroups were compared using the Mann–Whitney U test, and categorical variables were compared using Pearson’s chi-square or Fisher’s exact tests, as appropriate. We used mean imputation for missing data.

The occurrences of KDIGO stage 2, stage 3 AKI, and newly onset dialysis were compared between patients treated with Locolin® and those treated with Colimycin®. Kaplan–Meier curves were constructed to evaluate the difference in the occurrence of AKI between subgroups of patients. Cox regression analysis was performed to identify independent variables associated with KDIGO 3 AKI. Treatment outcomes including mechanical ventilator days, ICU stays, hospital stays, and all-cause mortality were also compared between patients with and without the occurrence of KDIGO stage 3 AKI. All tests were two-tailed, and a *p*-value of < 0.05 was considered statistically significant.

## Results

### Patient characteristics

**Fig. 1:**
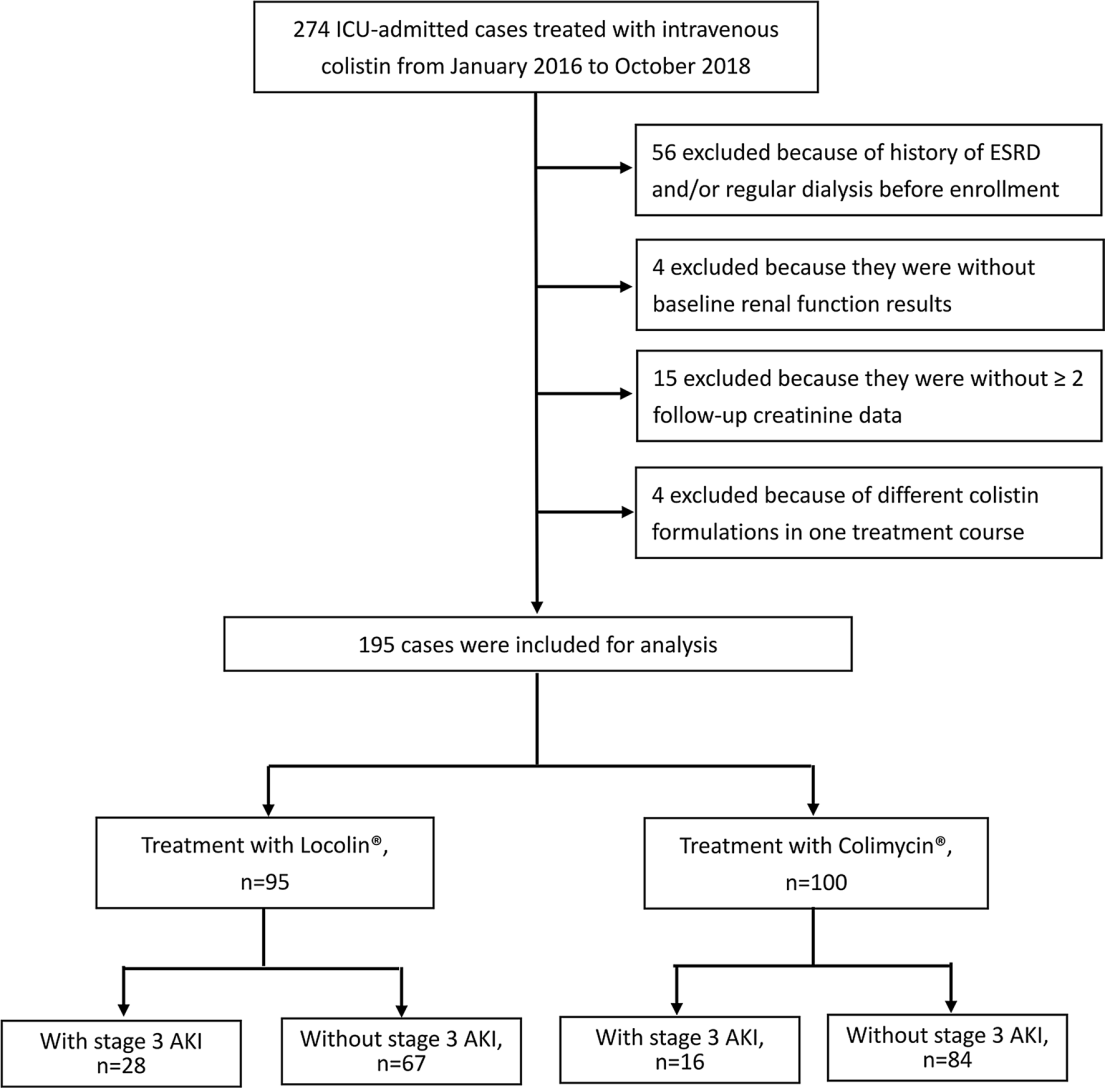
Study flow diagram and reasons for patient exclusions. ESRD, end stage renal diseases; AKI, acute kidney injury

During the study period, the medical records of 195 ICU-admitted patients who were administered with intravenous colistin were analyzed. Among these patients, 95 received Locolin® and 100 received Colimycin®. A flowchart of the number of cases and reasons for exclusion is shown in Fig. 1. The demographic characteristics and disease severities of the enrolled patients are summarized in Table 1. Mean age of the enrolled patients was 73.8 ± 13.5 years old and 70.8% (138/195) were male. Diabetes is the most common comorbidity (57/195, 29.2%) and 10.8% (21/195) of the enrolled cases had renal insufficiency. The median APACHE II score of the patients at ICU admission was 24 (IQR 19–30). At the time of colistin administration, 89.7% (175/195) had respiratory failure and 31.3% (61/195) had septic shock. Approximately 80% of the CR-GNB isolates were cultured from respiratory specimens. The proportions of CRAB, CRE, and CRPA were 72.3%, 23.1%, and 4.6%, respectively. The median daily colistin maintenance dose was 8 MIU (IQR 4–10), and the median treatment duration of colistin was 7 days (IQR 4–12). Twenty-three patients (11.8%) received colistin at an inappropriate maintenance dosage. Comparatively, patients who received Locolin® had a higher serum albumin level (2.8 g/dL vs. 2.6 g/dL, p = 0.017) and were more likely to receive concomitant aminoglycoside treatment (15.8% vs. 7%, p = 0.052) and less likely to have colistin at an inappropriate dosage (7.4% vs. 16%, p = 0.062) than those who received Colimycin®. Only few patients had concomitant non-steroid anti-inflammatory drugs (n = 6), and no patient had concomitant amphotericin B (data not shown). Otherwise, the two groups of patients had similar demographic characteristics, underlying comorbidities, and disease severities. There were no significant differences in the dosage and duration of colistin treatment between the two groups of patients.

**Table 1:** Demographic characteristics of ICU-admitted patients treated with two different formulations of intravenous colistin^a^

	Overall patients	Intravenous colistin		P-value
		Locolin®	Colimycin®	
No. of patients	195	95	100	
Age, years (SD)	73.8 (13.5)	75.2 (12.3)	72.4 (14.6)	0.142
*Sex*				0.069
Male	138 (70.8%)	73 (76.8%)	65 (65%)	
Female	57 (29.2%)	22 (23.2%)	35 (35%)	
BMI (SD)	22.3 (4.6)	21.7 (4.8)	22.8 (4.3)	0.106
Smoking	78 (40%)	40 (42.1%)	38 (38%)	0.559
*Comorbidities*				
Diabetes	57 (29.2%)	28 (29.5%)	29 (29%)	0.942
Malignancies	33 (16.9%)	24 (25.3%)	9 (9%)	0.002
Renal insufficiency (CCr < 30)	21 (10.8%)	10 (10.5%)	11 (11%)	0.915
Chronic liver diseases	16 (8.2%)	3 (3.2%)	13 (13%)	0.017
Heart failure	17 (8.7%)	8 (8.4%)	9 (9%)	0.886
*CR-GNB culture sources*				
Respiratory specimens	157 (80.5%)	75 (78.9%)	82 (82%)	0.591
Urine	11 (5.6%)	6 (6.3%)	5 (5%)	0.691
Blood	10 (5.1%)	4 (4.2%)	6 (6%)	0.571
Others	17 (8.7%)	10 (10.5%)	7 (7%)	0.383
*CR-GNB species*				
CRAB	141 (72.3%)	64 (67.4%)	77 (77%)	0.270
CRE	45 (23.1%)	28 (29.5%)	17 (17%)	0.051
CRPA	9 (4.6%)	3 (3.2%)	6 (6%)	0.344
*Laboratory results (Mean, SD)*				
Leukocytes (× 10^9^ per L)	13.2 (9.0)	13.2 (8.3)	13.3 (9.7)	0.935
CRP (mg/dL)	10.0 (6.5)	10.3 (7.6)	9.6 (5.9)	0.464
Albumin level (median, IQR)	2.7 (2.4–3.0)	2.8 (2.5–3.1)	2.6 (2.3–2.9)	0.017
*Concomitant nephrotoxins*				
Aminoglycoside	22 (11.3%)	15 (15.8%)	7 (7%)	0.052
Vancomycin	20 (10.3%)	13 (13.7%)	7 (7%)	0.124
Intravenous contrast agent	39 (20.0%)	17 (17.9%)	22 (22%)	0.474
*Disease severity*				
APACHEII scores (median, IQR)^b^	24 (19–30)	25 (20–30)	24 (19–30)	0.768
Respiratory failure^d^	175 (89.7%)	83 (87.4%)	92 (92%)	0.287
Septic shock^d^	61 (31.3%)	33 (34.7%)	28 (28%)	0.310
*Intravenous colistin*				
Daily dosage (MIU) (median, IQR)	8 (4–10)	8 (4–10)	8 (4–10)	0.814
Treatment duration (median, IQR)	7 (4–12)	7 (4–12)	7 (4–12)	0.782
Accumulated dosage (MIU) (median, IQR)	48 (28–80)	48 (28–64)	48 (24–88)	0.956
Inappropriate colistin dosage	23 (11.8%)	7 (7.4%)	16 (16%)	0.062

*APACHE II* Acute Physiology and Chronic Health Evaluation II, *BMI* body mass index, *ICU* intensive care unit, *IQR* interquartile range, *CCr* creatinine clearance, *CR-GNB* carbapenem-resistant gram-negative bacteria, *SD* standard deviation

^a^Data are presented as n (%)

^b^Including abscess, ascites, CSF, and pericardial effusion

^c^Evaluated on the day of ICU admission

^d^Present on the day of sample collection

### Rate of occurrence of AKI

**Fig. 2:**
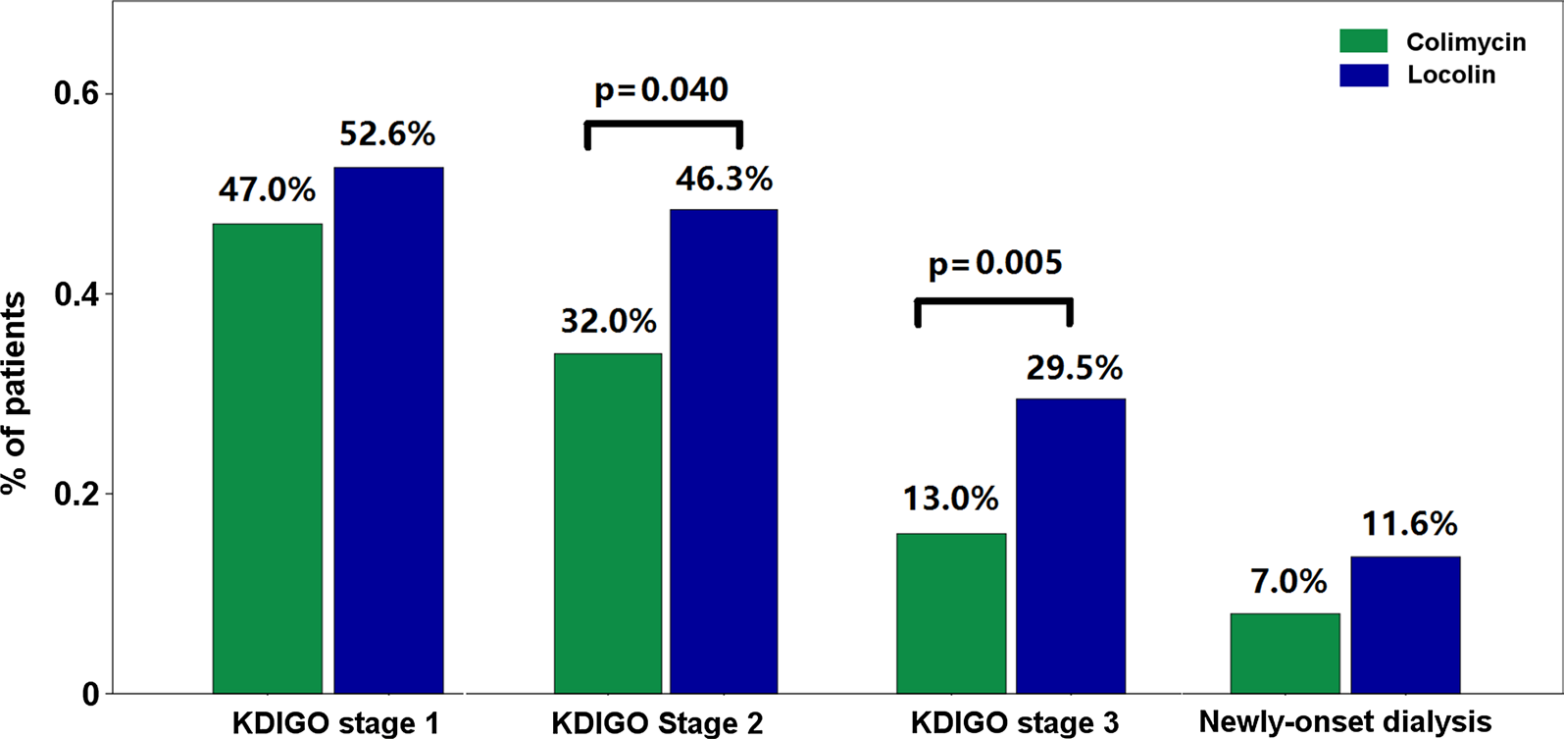
Rate of occurrence of acute kidney injury in ICU-admitted patients treated with intravenous colistin. KDIGO stage 1, stage 2, stage 3 AKI, and newly initiated dialysis are presented

The rates of occurrence of various severities of AKI and newly initiated dialysis within 28 days after colistin administration are shown in Fig. 2. Overall, the rate of occurrence of KDIGO stage 1, stage 2, stage 3 AKI, and newly initiated dialysis was 49.7%, 39%, 21%, and 9.2%, respectively. Comparatively, patients who received Colimycin® had a significantly lower rate of occurrence of KDIGO stage 2 AKI (32% vs. 46.3%, p = 0.040) and KDIGO stage 3 AKI (13% vs. 29.5%, p = 0.005) than those who received Locolin®. The rate of occurrence of newly initiated dialysis was comparable between the two groups of patients.

**Fig. 3:**
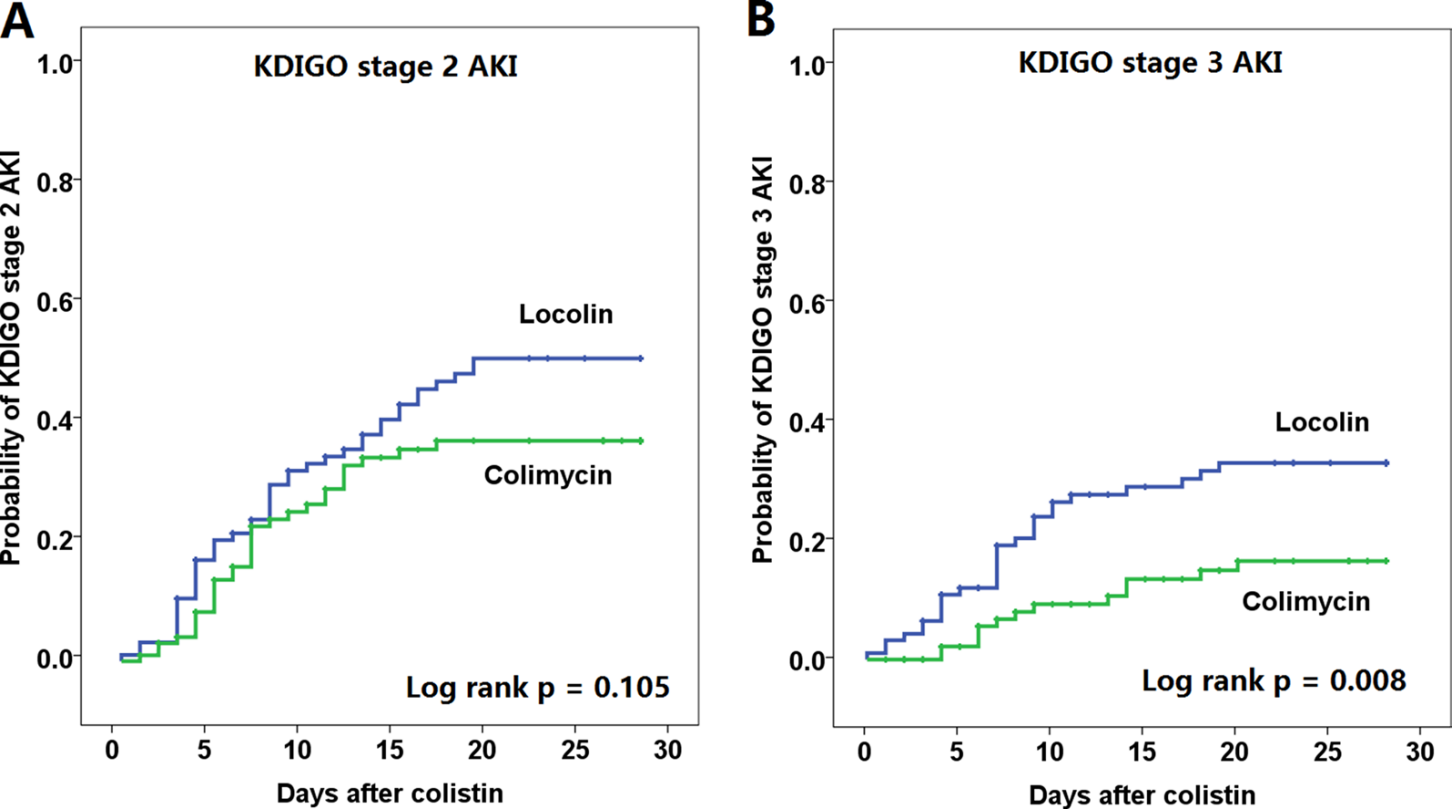
Kaplan–Meier analysis of **a** stage 2 AKI and **b** stage 3 AKI between patients treated with Locolin® and those treated with Colimycin®. AKI, acute kidney injury

Curves of Kaplan–Meier analysis of the occurrence of KDIGO stage 2 and stage 3 AKI in both groups of patients are shown in Fig. 3. Patients who received Colimycin® had a significantly lower rate of occurrence of KDIGO 3 AKI than those who received Locolin® (log rank p = 0.008). The curves separated early after the onset of colistin treatment.

Among the patients with KDIGO stage 3 AKI, the complete or partial recovery rates was 53.6% (15/28) in the Locolin® group and 53.8% (7/13) in the Colimycin® group. The median duration of AKI among patients with Locolin® and Colimycin® was 9 (IQR 7–13) days and 10 (IQR 8–12) days, respectively. Both AKI recovery rate and AKI duration between the colistin groups showed no significant differences.

### Clinical factors associated with AKI

Among the total enrolled patients, 41 (2%) had KDIGO stage 3 AKI. Comparisons of clinical factors between patients with and without KDIGO stage 3 AKI are shown in Table 2. Patients with KDIGO stage 3 AKI were more likely to have CR-GNB cultured from respiratory specimens (95.1% vs. 76.6%, p = 0.035), concomitant vancomycin treatment (19.5% vs. 7.8%, p = 0.028), septic shock (53.7% vs. 25.3%, p = 0.001), higher daily colistin dose (8 MIU, IQR 7.4–10 MIU vs. 8 MIU, IQR 4–10 MIU, p = 0.036), and inappropriate colistin maintenance dosage (22% vs. 9.1%, p = 0.023).

**Table 2:** Clinical characteristics of ICU patients with and without the occurrence of KDIGO 3 acute kidney injury^a^

	≥ KDIGO 3 AKI		P-value
	Yes, n = 41	No, n = 154	
Age, years (SD)	71.4 (12.2)	74.4 (13.8)	0.138
*Sex*			0.421
Male	26 (63.4%)	112 (72.7%)	
Female	15 (36.6%)	42 (27.3%)	
BMI (SD)	22.2 (5.6)	22.3 (4.3)	0.788
Smoking	15 (36.6%)	63 (40.9%)	0.363
*Comorbidities*			
Diabetes	12 (29.5%)	45 (29.2%)	0.958
Malignancies	10 (24.4%)	23 (14.9%)	0.243
Renal insufficiency	6 (14.6%)	15 (9.7%)	0.211
Chronic liver diseases	3 (7.3%)	13 (8.4%)	1.000
Heart failure	3 (7.8%)	14 (9.1%)	0.767
*CR-GNB culture sources*			0.035
Respiratory specimens	39 (95.1%)	118 (76.6%)	
Urine	0	10 (6.5%)	
Blood	2 (4.9%)	9 (5.8%)	
Others^b^	0	17 (11.0%)	
*CR-GNB species*			0.229
CRAB	34 (82.9)	107 (69.5%)	
CRE	6 (14.6%)	39 (25.3%)	
CRPA	1 (2.4%)	8 (5.2%)	
*Laboratory results (Mean, SD)*			
Leukocytes (× 10^9^ per L)	11.5 (9.9)	13.7 (8.8)	0.183
CRP (mg/dL)	10.5 (10.0)	9.8 (5.6)	0.555
Albumin level	2.8 (2.5–3.1)	2.7 (2.4–3.0)	0.217
*Concomitant nephrotoxins*			
Aminoglycoside	5 (12.2%)	17 (11.0%)	0.835
Vancomycin	8 (19.5%)	12 (7.8%)	0.028
Intravenous contrast agent	9 (22%)	30 (19.5%)	0.725
*Disease severities*			
APACHEII scores (median, IQ R)^c^	23 (18–31)	25 (20–30)	0.454
Respiratory failure^d^	40 (97.6%)	135 (87.7%)	0.081
Septic shock^d^	22 (53.7)	39 (25.3%)	0.001
*Colistin treatment*			
Daily dose (MIU) (median, IQR)	8 (7.4–10)	8 (4–10)	0.036
Treatment duration (days) (median, IQR)	7 (4–11)	7 (4–12)	0.403
Accumulated dose (MIU) (median, IQR)	46.5 (30.5–87.2)	48 (24–80)	0.594
Inappropriate colistin dose	9 (22.0%)	14 (9.1%)	0.023

*APACHE II* Acute Physiology and Chronic Health Evaluation II, *BMI* body mass index, *ICU* intensive care unit, *IQR* interquartile range, *CCr* creatinine clearance, *CR-GNB* carbapenem-resistant gram-negative bacteria, *SD* standard deviation

^a^Data are presented as n (%)

^b^Including abscess, ascites, CSF, and pericardial effusion

^c^Evaluated on the day of ICU admission

^d^Present on the day of sample collection

Results of univariate and multivariate analyses of the clinical factors associated with the occurrence of KDIGO stage 3 AKI are shown in Table 3. In multivariate analysis, the independent factors associated with KDIGO stage 3 AKI included septic shock (adjusted odds ratio [aOR] 2.17, 95% confidence interval [CI] 1.10–4.26) and inappropriate colistin dosage (aOR 2.52, 95% CI 1.00–6.33). By contrast, when compared to Locolin®, Colimycin® treatment was an independent factor associated with a lower risk of KDIGO stage 3 AKI (aOR 0.37, 95% CI 0.18–0.77).

**Table 3:** Univariate and multivariate Cox-regression analysis of clinical factors associated with the occurrence of KDIGO 3 acute kidney injury

	Univariate analysis		Multivariate analysis	
	OR (95% CI)	P-value	aOR (95% CI)^a^	P-value
Age	0.99 (0.97–1.01)	0.236	0.99 (0.96–1.02)	0.463
*Sex*				
Female	1.00	–	1.00	–
Male	0.72 (0.38–1.36)	0.312	0.70 (0.34–1.46)	0.346
Baseline eGFR < 30	1.65 (0.69–3.91)	0.260	1.41 (0.48–4.14)	0.536
Diabetes	0.93 (0.47–1.82)	0.824	1.16 (0.56–2.41)	0.681
APACHII ≥ 25	0.64 (0.34–1.20)	0.162	0.53 (0.24–1.17)	0.118
Albumin ≤ 3 g/dL	0.81 (0.42–1.54)	0.522	0.66 (0.34–1.28)	0.221
CRAB	0.61 (0.29–1.29)	0.197	0.53 (0.24–1.17)	0.118
Aminoglycoside	1.01 (0.40–2.58)	0.982	0.99 (0.34–2.86)	0.979
Vancomycin	1.99 (0.92–4.3)	0.082	1.59 (0.65–3.89)	0.310
Intravenous contrast	1.07 (0.51–2.23)	0.865	0.89 (0.37–2.12)	0.785
Septic shock^b^	2.79 (1.51–5.16)	0.001	2.17 (1.10–4.26)	0.025
Daily colistin > 10 MIU	1.75 (0.94–3.26)	0.077	1.32 (0.61–2.83)	0.482
Colistin treatment duration	0.98 (0.92–1.03)	0.411	0.99 (0.93–1.04)	0.617
Inappropriate colistin dosage	2.29 (1.09–4.80)	0.028	2.52 (1.00–6.33)	0.049
*Colistin formulation*				
Locolin®	1.00	–	1.00	–
Colimycin®	0.42 (0.22–0.82)	0.011	0.37 (0.18–0.77)	0.008

^a^Adjusted odds ratio (aOR) and 95%CI were derived from cox regression analysis

^b^Present on the day of sample collection

### Effect of AKI on treatment outcomes

We further explored the impact of KDIGO stage 3 AKI on treatment outcomes. As shown in Table 4, patients with KDIGO stage 3 AKI had longer ventilator using days (36 days, IQR 17–52 days vs. 22 days, IQR 13–42 days, p = 0.036). Otherwise, patients with and without KDIGO stage 3 AKI had comparable hospital stays, ICU stays, and all-cause mortality. Comparisons of treatment outcomes between patients receiving Locolin® and those receiving Colimycin® were also analyzed. As shown in Table 4, patients who were treated with Colimycin® were associated with shorter ventilator using days (28 days, IQR 15–48 days vs. 24 days, IQR 13–47 days, p = 0.048).

**Table 4:** Treatment outcomes of patients with and without KDIGO 3 acute kidney injury, and with two different formulations of intravenous colistin

	KDIGO 3 AKI		P-value	Intravenous colistin		
	Yes, n = 41	No, n = 154		Locolin®, n = 95	Colimycin®, n = 100	
Ventilator days (Median, IQR)	36 (17–52)	22 (13–42)	0.036	28 (15–48)	24 (13–47)	0.048
ICU stay (days) (Median, IQR)	31 (19–47)	30 (20–51)	0.889	30 (19–47)	31 (21–54)	0.285
Hospital stay (days) (Median, IQR)	60 (38–94)	53 (31–80)	0.133	56 (35–87)	50 (29–76)	0.075
*Mortality*						
Day 28	14 (34.1%)	62 (40.3%)	0.476	34 (35.8%)	42 (42%)	0.374
Discharge	28 (68.3%)	91 (59.1%)	0.283	56 (58.9%)	63 (63%)	0.562

*AKI* acute kidney injury, *ICU* intensive care unit, *IQR* interquartile range

Composition between Locolin® and Colimycin®

**Fig. 4:**
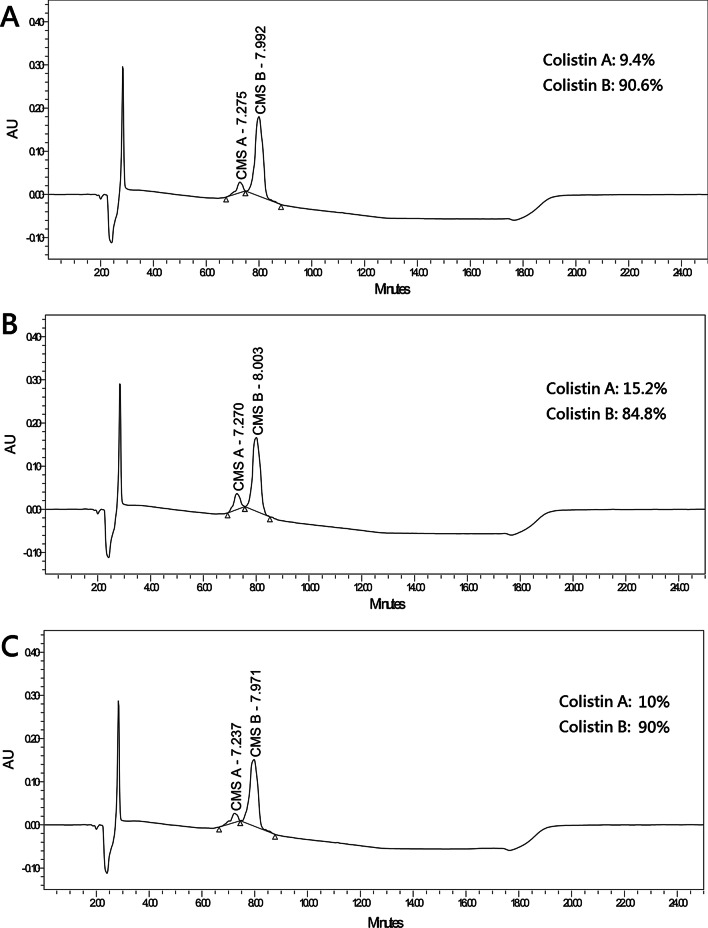
HPLC analysis for **a** CMS reference standard of CMS, **b** Locolin®, and **c** Colimycin®. The compositions of CMS A and CMS B are presented separately. *HPLC* high-performance liquid chromatography, *CMS* colistin methanesulfonate

The chromatographic profiles of Locolin®, Colimycin®, and reference standard are shown in Fig. 4. The proportions of CMS A in Locolin®, Colimycin®, and reference standard were 15.2%, 10%, and 9.4%, respectively.

## Discussion

This retrospective study enrolled critically ill patients who underwent intravenous colistin treatment for CR-GNB and evaluated the occurrence of AKI. During the colistin treatment period, KDIGO stage 1, stage 2, and stage 3 AKI occurred in 49.7%, 39%, and 21% of patients, respectively. Meanwhile, 9.2% of the patients had newly initiated dialysis. Comparatively, patients who received Colimycin® had a lower rate of occurrence of KDIGO stage 2 and stage 3 AKI than those who received Locolin®. In multivariate analysis, we found that independent factors associated with KDIGO stage 3 AKI included the presence of septic shock and inappropriate colistin dosage. By contrast, Colimycin® use was an independent factor associated with a lower rate of occurrence of KDIGO stage 3 AKI. We also found that the occurrence of KDIGO stage 3 AKI during colistin treatment was associated with longer mechanical ventilator using days but not related to higher all-cause mortality.

Nephrotoxicity and neurotoxicity are well-documented adverse reactions associated with intravenous treatment with colistin. Nephrotoxicity in colistin is dose-dependent and usually reversible [16, 18, 25, 26]. The nephrotoxicity of colistin is mainly related to its D-aminobutyric acid and fatty acid component. Apart from its bactericidal effects, colistin increases the membrane permeability of tubular epithelial cells, which, in turn, leads to cell swelling and lysis [10]. Concerning toxicity, colistin is administered as an inactive prodrug, CMS, which is the only parenteral form used clinically for colistin. CMS is off patent for many years, and several commercially available parenteral products of CMS are available on the market. However, the number and location of methane sulfonate groups attached on CMS vary widely in different colistin products, which may, in turn, lead to differences in pharmacokinetics and pharmacodynamics of colistin in humans [27, 28]. Limited studies have been performed to evaluate the differences between various brands of colistin. Li et al. reported a 20% difference in the colistin level in plasma between different formulations of colistin [27]. He et al. reported distinct chromatographic profiles of different colistin products [19]. They also demonstrated significantly different exposure to colistin between various brands of colistin in a rat model [19]. However, the differences in nephrotoxicity between various formulations of colistin remain unknown.

Colistin is a multicomponent lipopeptide that contains CMS A and CMS B, which differ in the fatty acid chain attached to the cyclic decapeptide moiety of the drug [29]. The proportion of CMS A and CMS B can have a large difference in commercial preparations of colistin [30]. Although there are comparable bactericidal effects between CMS A and CMS B, CMS A has been reported to have a higher nephrotoxic effect than CMS B in an animal model study [29]. The different compositions of CMS A and CMS B in various formulations of colistin might lead to different risks of colistin-induced nephrotoxicity. In the present study, we demonstrated a significant difference in the rate of occurrence of AKI between two different formulations of colistin. Furthermore, we reported that the proportion of CMS A in Locolin® is 50% higher than that in Colimycin®, which is in line with our clinical observation of an increased risk of nephrotoxicity in Locolin®. To the best of our knowledge, this is the first study to evaluate the occurrence of nephrotoxicity between different formulations of colistin. Although the exact mechanisms remain uncertain, we speculate that the difference in the composition of CMS A and CMS B in various colistin products could play a pivotal role. Clinicians should therefore be aware of the possible difference in the risks of nephrotoxicity among various formulations of colistin. Further studies are also warranted to verify our findings.

We further evaluated the impact of the occurrence of colistin-related AKI on treatment outcomes, which have rarely been evaluated thus far. We found that patients with colistin-induced AKI may have prolonged dependence on mechanical ventilator; there were no differences in mortality and hospital stays between patients with and without colistin-related AKI. Our findings were consistent with those of a previous study, which prospectively enrolled patients infected by extensively drug-resistant *Acinetobacter baumannii* and treated by colistin [31]. However, ventilator dependence and hospital stays were noted in that study. A study on patients infected by drug-resistant *Pseudomonas aeruginosa* reported the presence of AKI as an independent factor associated with a high mortality rate [32]. Another study reported that patients who experienced AKI had a higher mortality rate if kidney function failed to return to the baseline level [6]. Although the findings remain controversial, we believe that close monitoring of renal function during colistin treatment and early discontinuation of colistin in patients with AKI are the best ways to reduce the effect of AKI on treatment outcomes in these critically ill patients.

There are some limitations to this study. First, as this was a retrospective study, the demographic characteristics and disease severities were not equal between patients treated with Colimycin® and those treated with Locolin®. Although we had performed multivariate analysis to adjust for the effects from clinical factors, our findings should be interpreted with caution. Second, all the enrolled patients had CR-GNB isolated from clinical specimens, and some of the patients may have colonization rather than true infection. However, the leukocyte count and the C-reactive protein level in the enrolled patients were much above the upper normal limits, which indicated that most of the enrolled patients had infection rather than colonization. Meanwhile, the effect of colonization on our analysis was limited because this study aimed to investigate colistin-induced nephrotoxicity, rather than treatment effectiveness. Third, exposure to concomitant nephrotoxins, including vancomycin, aminoglycosides, and contrast agent, was not rare in our patients. Therefore, the risk of colistin-induced nephrotoxicity could be overestimated. Fourth, we excluded patients with ESRD but included those with renal insufficiency; which may affect the development of AKI during colistin treatment. However, the proportion of patients with renal insufficiency was comparable between the two colistin groups, and we included renal insufficiency in our multivariate analysis. Finally, we enrolled critically ill patients who had ICU admission and high APACHE II scores. Most of them had respiratory failure, and nearly one-third of them received inotropic agents. Therefore, the findings obtained in our study may not be applicable to patients with low disease severities.

## Conclusions

This retrospective study involved critically ill patients who were treated with intravenous colistin. We found significant differences in the rate of occurrence of colistin-induced nephrotoxicity between two formulations of colistin. We also demonstrated different compositions in the two formulations of colistin. Other clinical factors associated with colistin-induced nephrotoxicity included septic shock and inappropriate colistin maintenance dosage. Our findings suggest that the risk of nephrotoxicity in colistin could be different in various formulations of colistin. The association between the risk of nephrotoxicity and the differences in the compositions of various colistin formulations deserves further studies for clarification. Meanwhile, close monitoring of renal function in high-risk populations and appropriate dosage adjustment during colistin treatment is crucial to decrease the risk of colistin-induced nephrotoxicity in critically ill patients.

## Supplementary Information

**Additional file 1**. Recommended loading dose and daily maintenance doses of colistimethate.

## Data Availability

The datasets used and/or analyzed during the current study are available from the corresponding author on reasonable request.

## Abbreviations

MDRO: Multidrug-resistant organisms
CR-GNB: Carbapenem-resistant gram-negative bacilli
CRAB: Carbapenem-resistant-Acinetobacter baumannii complex
CRE: Carbapenem-resistant Enterobacterales
CRPA: Carbapenem-resistant Pseudomonas aeruginosa
AKI: Acute kidney injury
ICU: Intensive care units
APACHE: Acute Physiology and Chronic Health Evaluation
eGFR: Estimated glomerular filtration rate
NAC: N-acetylcysteine
