# Supplementary material for: Comparison of colistin-induced nephrotoxicity between two different formulations of colistin in critically ill patients: a retrospective cohort study
Source: Antimicrob Resist Infect Control. 2021 Jul 30;10:111. doi: 10.1186/s13756-021-00977-w (PMC8323087; doi:10.1186/s13756-021-00977-w)
Supplement: Supplementary file 1 — Additional file 1. Recommended loading dose and daily maintenance doses of colistimethate. [file 13756_2021_977_MOESM1_ESM.docx]

**Supplementary Table 1**. Recommended loading dose and daily maintenance doses of colistimethate

|  | Dose suggestion (MIU) |
| --- | --- |
| Loading dose |  |
| Body weight (kg) |  |
| ≥ 60 | 9 |
| 50 | 7 |
| 40 | 6 |
| Daily maintenance dose |  |
| CrCl (mL/min/1.73 m^2^) |  |
| ≥ 70 | 10 |
| 50 | 8 |
| 20 | 4 |
| 0 | 2 |

MIU, million IU; CrCl creatinine clearance.
